# Expanded Functional Diversity of Shaker K^+^ Channels in Cnidarians Is Driven by Gene Expansion

**DOI:** 10.1371/journal.pone.0051366

**Published:** 2012-12-10

**Authors:** Timothy Jegla, Heather Q. Marlow, Bihan Chen, David K. Simmons, Sarah M. Jacobo, Mark Q. Martindale

**Affiliations:** 1 Department of Biology and Huck Institute of Life Sciences, Eberly College of Science, Penn State University, University Park, Pennsylvania, United States of America; 2 Kewalo Marine Laboratory, University of Hawaii, Honolulu, Hawaii, United States of America; 3 Department of Cell Biology, The Scripps Research Institute, La Jolla, California, United States of America; Virginia Commonwealth University, United States of America

## Abstract

The genome of the cnidarian *Nematostella vectensis* (starlet sea anemone) provides a molecular genetic view into the first nervous systems, which appeared in a late common ancestor of cnidarians and bilaterians. *Nematostella* has a surprisingly large and diverse set of neuronal signaling genes including paralogs of most neuronal signaling molecules found in higher metazoans. Several ion channel gene families are highly expanded in the sea anemone, including three subfamilies of the Shaker K^+^ channel gene family: Shaker (Kv1), Shaw (Kv3) and Shal (Kv4). In order to better understand the physiological significance of these voltage-gated K^+^ channel expansions, we analyzed the function of 18 members of the 20 gene Shaker subfamily in *Nematostella*. Six of the Nematostella Shaker genes express functional homotetrameric K^+^ channels *in vitro*. These include functional orthologs of bilaterian Shakers and channels with an unusually high threshold for voltage activation. We identified 11 Nematostella Shaker genes with a distinct “silent” or “regulatory” phenotype; these encode subunits that function only in heteromeric channels and serve to further diversify Nematostella Shaker channel gating properties. Subunits with the regulatory phenotype have not previously been found in the Shaker subfamily, but have evolved independently in the Shab (Kv2) family in vertebrates and the Shal family in a cnidarian. Phylogenetic analysis indicates that regulatory subunits were present in ancestral cnidarians, but have continued to diversity at a high rate after the split between anthozoans and hydrozoans. Comparison of Shaker family gene complements from diverse metazoan species reveals frequent, large scale duplication has produced highly unique sets of Shaker channels in the major metazoan lineages.

## Introduction

The classical view of cnidarian nervous system as a simple, diffuse nerve net has been eroded by recent anatomical and genetic findings. Sequencing of the Nematostella vectensis (anthozoan) and Hydra magnipapillata (hydrozoan) genomes revealed that the molecular building blocks of the nervous system (including genes that regulate neuronal development, synapse formation and electrical signaling) are conserved between cnidarians and bilaterians [1], [2]. The ion channel families shared between bilaterians and cnidarians were likely present in the first nervous systems and may represent a fundamental set of ion channels required for complex neuronal signaling. 43 of 46 ion channel families conserved across Bilateria, including all major classes of voltage-gated ion channels, are also present in *Nematostella* [3]. Voltage-gated channels play a key role in tuning the intrinsic excitability of neurons, including determining the shape, frequency and pattern of action potentials that can be elicited upon stimulation. In protostome invertebrate model organisms such as *Drosophila* and *Caenorhabditis elegans*, voltage-gated channel families are often represented by a single or only a few extant genes [3], [4]. Therefore, it was surprising that the cnidarians, which have traditionally been viewed as having simple nervous systems, had an extensive set of voltage-gated ion channel genes, including cnidarian-specific expansions in 5 of 14 ancestral K^+^ channel families [2], [3]. The large number of genes implies a high degree of diversity in the intrinsic electrical properties of cnidarian neurons and raises the question of why such diversity is needed in a comparatively simple nervous system.

Part of the answer to this question is undoubtedly that cnidarian nervous systems are more complex than originally thought. For instance, neural development in Nematostella appears to be driven by a complex cascade of highly conserved neuronal transcription factors [5]. The nervous system has 7 distinct classes of cells that can be identified with simple histological labels [5]. While generally diffuse, the Nematostella polyp nervous system has two well-defined nerve rings, oral and pharyngeal, which contain significant concentrations of neurons; tentacles are also highly innervated [5]. Nerve rings or ganglia are a common feature of cnidarians, and their connections with sensory and motor systems are highly organized and specialized [6], [7], [8], [9]. These features coordinate contraction to produce a host of complex behaviors such as, slow swimming and escape responses, cross-talk between feeding behavior and swimming, capture of prey and regulation of movement by sensory stimuli such as light and touch [7], [8], [9], [10], [11]. Producing complex behaviors from a small nervous system may require a set of neurons with diverse intrinsic electrical properties, which could in part be provided by an extensive and diverse ion channel set.

In order to better understand the physiological role of voltage-gated ion channel diversity in cnidarians, we functionally characterized the highly expanded *Nematostella* Shaker, or Kv1, potassium channel subfamily. The Drosophila Shaker gene was the first K^+^ channel cloned [12], [13] and is the archetypal representative of the Shaker family of voltage-gated K^+^ channels which includes four closely related gene subfamilies (Shaker (Kv1), Shab (Kv2), Shaw (Kv3) and Shal (Kv4)) [4], [14]. Potassium channels assemble as tetramers [15] with a single K^+^-selective pore; Tetrameric assembly in Shaker family channels is promoted by a unique N-terminal cytoplasmic domain, T1 [16], [17]. Heterotetramers can form between members of the same gene subfamily, but not across subfamilies due primarily to cross-subfamily incompatibility of T1 domains [16], [17], [18]. Thus the four Shaker-related subfamilies encode functionally independent suites of voltage-gated K^+^ channels [18]. All previously characterized Shaker subfamily genes encode subunits can form functional homotetrameric channels *in vitro*. Bilaterian Shaker subfamily channels characteristically activate near action potential threshold with rapid kinetics and are thus well suited to regulate action potential threshold and repolarization [19], [20], [21], [22]. Two cnidarian Shaker subfamily channels from the hydrozoan Polyorchis penicillatus, jShak1 and jShak2, show functional orthology to bilaterian Shakers in terms of activation rate and inactivation mechanisms, but have unusually high voltage-activation thresholds [23]. We show here that the large Nematostella Shaker family provides a remarkable diversity of Shaker currents, including functional orthologs of bilaterian Shaker channels, cnidarian-specific high threshold Shakers orthologous to jShak1 and jShak2, and unique “regulatory” subunits that further diversify voltage-dependent gating properties. Regulatory K^+^ channel subunits function only in heteromeric channels and have previously been identified in the mammalian Shab and Polyorchis Shal subfamilies [24], [25], [26]. Many Nematostella Shaker genes are expressed contemporaneously in polyps, suggesting diverse electrical properties in excitable cells.

## Materials and Methods

### Ethics Statement

Nematostella vectensis husbandry was carried out according to best practices developed in the Nematostella community to optimize animal health.

### Gene Identification and Cloning

Shaker family gene complements from mouse, human, *Drosophila melanogaster* and *Caenorhabditis elegans* have been well established and were downloaded from RefSeq [27]. Subsets of these sequences were used as queries in BLAST searches [28] to identify the full complement of Shaker family genes in genomes drafts from *Nematostella vectensis* (Cnidaria, Anthozoa) [2], *Hydra magnipapillata* (Cnidaria, Hydrozoa) [1], *Trichoplax adhaerens* (Placozoa) [29], *Caenorhabditis briggsae* (Nematoda) [30], *Helobdella robusta* (Annelida) [31], *Capitella teleta* (Annelida) [31], *Lottia gigantea* (Mollusca) [31], *Daphnia pulex* (Arthropoda) [32], *Anopheles gambiae* (Arthropoda) [33], *Strongylocentrotus purpuratus* (Echinodermata) [34] and *Branchiostoma floridiae* (amphioxus, Chordata) [35]. Genomes were first searched using TBLASTN with verified Shaker family amino acid sequences to identify candidate loci. Draft protein predictions associated with these loci were used in further analysis in most cases. Protein predictions were manually assembled in the absence of a protein annotation or if a protein annotation included obvious errors. Only sequences that shared reciprocal best matches to Shaker family channels in BLASTP [28] comparisons to RefSeq [27] were considered to represent Shaker family genes. Five cloned and verified *Polyorchis penicillatus* (Hydrozoa) Shaker family sequences were also included in phylogenetic analysis ([23], [24], T. Jegla, Ph.D. diss., Washington University, 1996).

*Nematostella* Shaker subfamily coding sequences were cloned by either RT-PCR or genomic PCR (for some intronless sequences) using standard techniques. Multiple independent clones were fully sequenced for each gene to verify the coding sequence. Predicted amino acid sequences from verified sequences are included in Table S1, and DNA sequences along with GenBank accession numbers are provided in Table S2. Truncated versions of NvShak1 (Δ2–30), NvShak4 (Δ2–48), NvShak5 (Δ2–58) and NvShak6 (Δ1–16) were constructed using standard PCR techniques. Clones were sequence verified prior to expression and deletion numbers refer to amino acid residue positions.

### Expression in Xenopus Oocytes and Data Analysis

Clones of full coding sequences were shuttled to the pOX expression vector [24] for expression in Xenopus oocytes. Capped, poly-adenylated transcripts were prepared from linearized expression plasmids using T3 mMessage Machine and PolyA tailing kits (Life Technologies, Grand Island, NY). Transcripts were cleaned by LiCl precipitation and resuspended in nuclease free water supplemented with a 1∶20 dilution of RNAse Inhibitor (Life Technologies) to prevent degradation during handling. 1–100 ng of purified transcript were injected in 50 nl volumes into mature, enzymatically defolicullated Xenopus oocytes and recordings were made 1–4 days after injection. High transcript concentrations were injected for excised patch studies and low concentrations were injected for whole cell studies. For Co-expression, regulatory subunit transcripts were mixed with NvShak3 at a ratios ranging from 1∶1 to 10∶1. Xenopus oocytes were obtained from Nasco (Fort Atkinson, WI).

Whole cell two-electrode voltage clamp (TEV) currents were recorded using a Dagan CA–1B amplifier (Minneapolis, MN) and the pClamp acquisition suite (Molecular Devices, Sunnyvale, CA). Currents were leak subtracted using P/N protocol to remove linear leak (less than 50 nA) and capacitive transients. Recordings were carried out under constant perfusion of a low Cl^-^ solution (98 mM Na^+^, 2 mM K^+^, 1 mM Mg^2+^, 1 mM Ca^2+^, 5 mM HEPES, 4 mM Cl^-^, 100 mM Methanesulfonate (MES), pH 7.2). Bath clamp circuitry was isolated using a 1 M NaCl-agarose bridge. Electrodes were filled with 3 M KCl and had resistances of 0.4–1 MΩ.

For excised inside out patch recordings, oocytes were first shrunk in a hypertonic stripping solution [36], and vitelline envelopes were removed with forceps. Oocytes were bathed with internal solution (136 mM KMES, 4 mM KCl, 5 mM EGTA, 10 mM HEPES, pH 7.2), and excised patches were moved directly into a focal stream of this internal solution. Standard pipette (external) solution consisted of 138 mM NaMES, 2 mM NaCl, 2 mM KCl, 0.2 mM CaCl2, 10 mM HEPES, pH 7.2). For experiments conducted in symmetrical K^+^, NaMES was replaced with KMES. Grounds were bridged as described for TEV. Currents were recorded using a Multiclamp 700B amplifier and the pClamp acquisition suite (Molecular Devices, Sunnyvale, CA). Pipette capacitance and series resistance were compensated, and bath offsets were cancelled prior to seal formation. Patch pipettes were coated with Sticky Wax (Kerr Laboratories, Romulus, MI) to reduce capacitance and fire polished to a resistance of 0.6–1.5 MΩ.

Boltzmann fits of voltage activation and steady state inactivation data from individual cells used the equation g(V) = (A_1_-A_2_)/(1+ *e* ^((V-V50)/s)^) + A_2_, where g(V) is the conductance at voltage V, *V_50_* is the half-maximal conductance value, *s* is the slope factor and *A_1_* and *A_2_* are the minimum and maximum, respectively. Normalized data are displayed as mean ± S.E.M. with a simulated Boltzmann fit (G/G_max_ = 1/(1+ e^-((V-V50)/s)^) in which V_50_ and *s* are fixed to the mean values obtained from fits of data from individual cells. Single exponential fits of the late phase of activation (defined as the last 50% of current rise) and tail current decay used the equation I(t) = I_i_+A*e* ^-t/Τ^, where I(t) is the current at time t, I_i_ is the initial current, A is the amplitude of the fit and Τ is the time constant. Sigmoidal delay of activation was quantified as the time between the start of the voltage pulse and the time at which an exponential fit of the late phase of activation intercepted the zero current line. Exponential fitting was carried out in Clampfit (Molecular Devices) and Boltzmann fitting was carried out in Origin (OriginLab, Northampton, MA).

### *In situ* Hybridization

Fixation of polyps, hybridization procedures and probe detection were carried out as previously described [37], [38]. Briefly, Nematostella polyps were relaxed in 7% MgCl2 mixed 1∶1 with 1/3x FSW and fixed in 3.7% formaldehyde, 0.3% glutaraldehyde in 1/3 X seawater. Digoxygenin-labeled riboprobes of full coding regions were transcribed using the Megascript kit (Life Technologies, Grand Island, NY) and diluted to a final concentration of 1 ng/µL for hybridization. Hybridization was carried out for 1–2 days at 65°C, specimens were extensively rinsed and probes were detected colorimetrically using an alkaline phosphatase conjugated antibody and the substrate nitroblue tetrazolium-5-bromo-4-chloro-3-indoyl phosphate, p-toludine salt. Specimens were washed 5 times in PTw (standard phosphate buffered saline (PBS) supplemented with 0.1% tween20) and mounted in 70% glycerol in PBS. DIC imaging was performed on a Zeiss Axiophot microscope under a 20× objective. Photos were taken with a Zeiss AxioCam HRc camera and brightness, contrast and color balance were adjusted in Photoshop.

### Phylogenetic Analysis

Shaker family channel sequences from various metazoans used in phylogenetic analysis were obtained from GenBank submissions, genome annotations or molecular cloning (Nematostella). Sequences used and source citations (where applicable) are given in Table S1. A few sequences gleaned from genome annotations were excluded from phylogenetic analysis due to large sequence gaps but are still included in Table S1. Alignments were generated using ClustalW as implemented in Mega 5 [39] and trimmed to remove areas of length polymorphism. Bayesian inference implemented in MrBayes [40] was used to construct the phylogeny following parameters: 2 independent runs of four chains were run for 1,056,000 generations under a mixed model. Trees were sampled once every 500 generations for a total of 2,111 trees per run. Upon completion of the analysis, the first 25% of trees were discarded and the resulting phylogeny was based on the consensus of the two runs.

## Results and Discussion

### Identification and Cloning of Nematostella Shaker Genes

We identified 20 Nematostella Shaker subfamily genes using mouse Shaker amino acid sequences to BLAST query [28] the Nematostella genome and predicted protein set [2]. All 20 Nematostella sequences shared reciprocal best matches to bilaterian Shaker subfamily channels in BLAST queries. We successfully cloned the full open reading frame for 18 of the 20 genes using a combination of genomic and RT-PCR from mixed stage animals. Partial clones were obtained by RT-PCR for the remaining genes, confirming that they are expressed. Amino acid translations are provided in Table S1 and DNA coding sequences and GenBank accession numbers are given in Table S2.

Examination of the genomic structure of the 18 complete *Nematostella* Shaker ORFs revealed 14 intronless gene loci (labeled in Table S2). Mammalian Shaker genes derive from vertebrate-specific gene duplications, and 7 of 8 are also intronless [41], [42]. A lone Shaker gene we identified in the genome of the basal chordate Strongylocentrotus purpuratus [34] (Table S1) was also intronless, suggesting that chordate and most cnidarian Shaker genes may have evolved from a common intronless ancestor. We were not able to clone splice variants for 6 intron-containing *Nematostella* Shaker genes, and sequence searches did not identify potential alternative exons in regions of conservation. Alternative splicing therefore does not appear to be an important mechanism for producing functional diversity of Shaker channels in either mammals or cnidarians. Protostome invertebrate Shaker genes, in contrast, can have complex intron/exon structures and functionally significant alternative splicing. *Drosophila* Shaker, for instance, is alternatively spliced to produce channels with distinct inactivation kinetics [43], [44]. Inactivation gating diversity in mammalian Shaker channels is instead produced with multiple genes and co-assembly with cytoplasmic β1-subunits [45]. We found no clear evidence for orthologs of Shaker β-subunits in BLAST searches of Nematostella (data not shown). Therefore, we hypothesized that functional diversity of Shaker channels in Nematostella must primarily be derived from extensive gene duplication and diversification.

### Functional Expression of NvShak1-6

**Figure 1:**
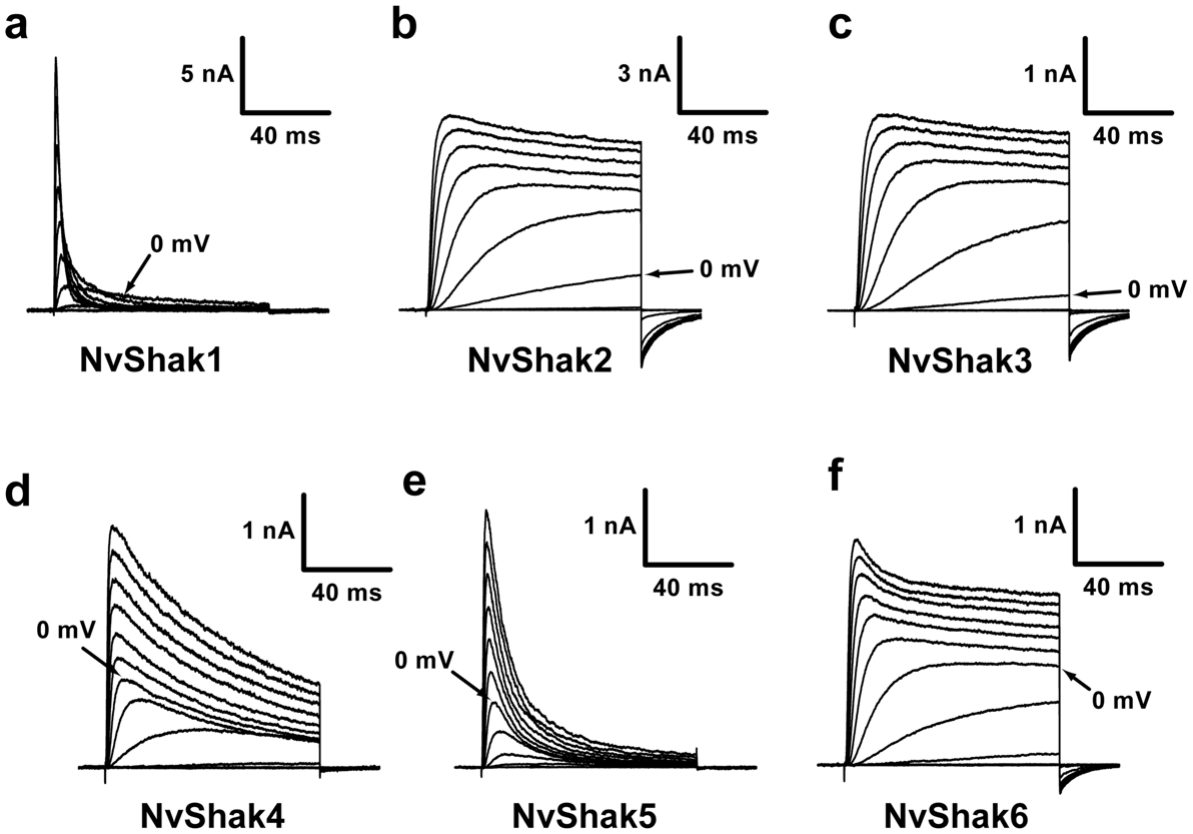
NvShak1-6 express a functionally diverse set of Shaker currents in *Xenopus* oocytes. Families of outward K^+^ currents recorded from excised inside-out patches taken from oocytes expressing NvShak1-6 are shown in a–f. Currents were recorded in response to 100 ms depolarizations ranging from −60 to +60 mV in 10 mV increments from a holding potential of −100 mV. Scale bars are given for time and current amplitude and arrows mark the current elicited at 0 mV. Note this is the first step with significant current for NvShak2 and NvShak3. K^+^ concentration for these recordings was 2 mM in the pipette (extracellular) and 140 mM in the bath (intracellular).

We tested functional expression of 18 full-length Nematostella Shaker genes in *Xenopus* oocytes. Surprisingly, only 6 of 18 genes, NvShak1-6, gave rise to voltage-gated K^+^ currents when expressed individually. Therefore, only these 6 genes encode subunits that can assemble into functional homotetramers. All previously expressed Shaker subunits, like NvShak1-6, can assemble into functional homotetrameric channels *in vitro* (although heterotetramers are known to exist in mammals *in vivo* [19], [21], [46]). We theorized that the remaining 12 channels might require heteromeric assembly, but we first examined the biophysical properties of NvShak1-6 in greater detail. Currents recorded from excised inside out patches are shown for NvShak1-6 in Figure 1. NvShak1-6 currents varied dramatically in inactivation kinetics. NvShak1 and NvShak5 inactivated fully within a few milliseconds, while NvShak4 and NvShak6 inactivated slowly or partially, respectively. NvShak2 and NvShak3 did not have significant inactivation and additionally had an apparent high activation threshold - current was only elicited by depolarization to −10 mV and above. This high threshold is similar to that observed for the Polyorchis penicillatus (cnidarian, Hydrozoa) Shaker channels jShak1 and jShak2 [23].

**Figure 2:**
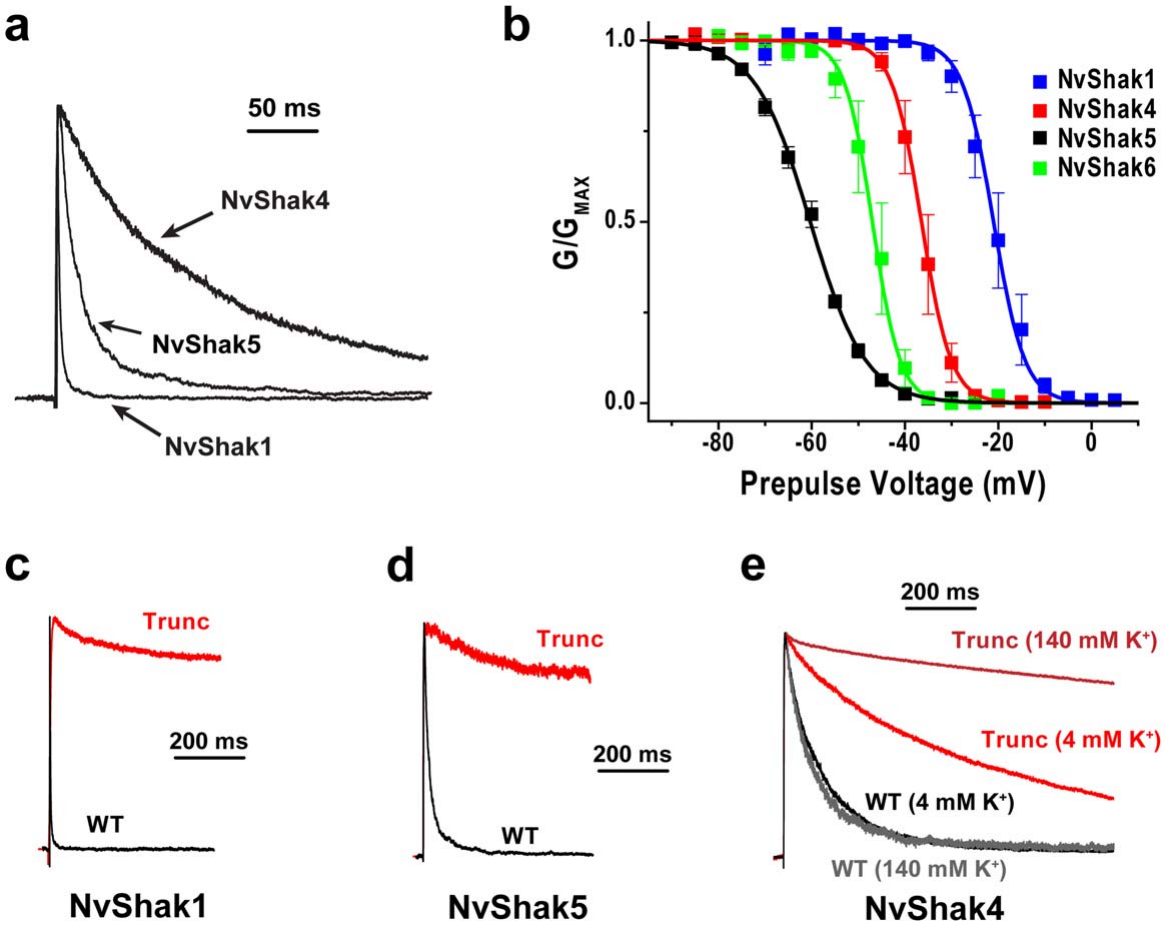
Comparison of inactivation in NvShak channels. ***(a)*** Comparison of inactivation time course in NvShak1, NvShak4 and NvShak5 currents recorded from excised patches in response to a depolarization to +60 mV. Currents were recorded in 2 mM external K^+^ and were normalized by peak amplitude to facilitate comparison of inactivation. Scale bar indicates time. ***(b)*** Steady state inactivation curves are given for NvShak1, NvShak4, NvShak5 and NvShak6. Data points show mean peak current ± S.E.M. (n = 4–6) elicited during a +60 mV test pulse following a 5 s pre-pulse to the indicated voltage. Data from individual patches were normalized in amplitude prior to comparison. Curves represent single Boltzmann distribution fits; V_50_ and slope values are reported in Table 1. Currents elicited at +60 mV for wild type (WT, black) and N-terminal truncated (Trunc, red) versions of NvShak1, NvShak5 and NvShak4 are compared in ***c-e***, respectively. Currents were normalized in amplitude for display. Truncations eliminated amino acid residues 2–30, 2–48 and 2–58 for NvShak1, NvShak4 and NvShak5, respectively. Truncation eliminated fast inactivation in NvShak1 and NvShak5, but only reduced the rate of inactivation in NvShak4. 140 mM K+ had little effect on the inactivation time course of WT NvShak4 (gray) but eliminated residual inactivation in the truncated form of NvShak4 (dark red), suggesting an unmasked C-type inactivation process in the truncated channel.

Comparison of NvShak1, NvShak4 and NvShak5 revealed an approximately 50-fold range in inactivation time course (Figure 2a, Table 1), spanning much of the observed diversity in inactivation rates observed across all Bilaterian Shaker channels. The rapid inactivation of NvShak1 is very similar to that of the *Drosophila* Shaker A and B splice variants [44], mammalian Shakers co-expressed with a β1-subunit [45], or jShak1 [23]. The inactivation time course of NvShak4, on the other hand, is similar to that of the mammalian Shaker channel Kv1.4 [47], the Drosophila Shaker H37 variant [43], and jShak2 [23]. Time constants for single exponential fits of inactivation in NvShak1, NvShak4 and NvShak5 are reported in Table 1. Several mammalian Shaker channels have little to no intrinsic inactivation (in the absence of a β1-subunit), as we observed for NvShak2 and NvShak3. Thus with respect to inactivation, Nematostella has functional orthologs of the major Shaker phenotypes present in bilaterians even in the absence of splicing or β-subunits. We examined steady state inactivation using pre-pulses to varying voltages to determine the voltage range over which the NvShak1,4,5 and 6 channels inactivate and become unresponsive to subsequent depolarization. Fractional availability of channels in the test depolarization is plotted vs. pre-pulse voltage in Figure 2b; V_50_ and slope values for steady state inactivation are given in Table 1. We found an ∼40 mV range in steady state V_50_ values, from −59 mV for NvShak5 to −20 mV for NvShak1, suggesting that these channels are tuned to respond in distinct voltage ranges.

**Table 1:** Voltage-dependent properties of NvShak1-6 Currents.

	τ_inact_ (ms)	SSI V_50_	SSI Slope	GV V_50_	GV Slope
**NvShak1**	2.4±0.2	−20.3±2.4	3.3±0.3	−19.5±0.5	5.7±0.3
**NvShak2**	N.A.	N.A.	N.A.	2.1±0.4	3.3±0.1
**NvShak3**	N.A.	N.A.	N.A.	−0.8±1.8	3.6±0.2
**NvShak4**	33.5±5.8 (.32)*153.6±20.3 (.68)*	−37.0±1.8	2.2±0.3	−27.4±1.5	2.2±0.2
**NvShak5**	14.1±1.1	−59.0±1.4	6.1±0.5	−30.1±0.6	4.3±0.4
**NvShak6**	N.A.	−46.2±1.6	3.1±0.3	−25.7±1.5	3.6±0.4

τ_inact_ is the time constant of inactivation at +60 mV derived from a single exponential fit.

* The inactivation time course of NvShak4 was best fit by two exponentials and both time constants are reported. Fractional amplitudes of each component are given in parentheses.

SSI refers to the steady state inactivation curve. Half-inactivation (V_50_) and Slope values of Boltzmann fits are reported in mV.

GV is conductance-voltage (or voltage activation) curve. Half-activation (V_50_) and Slope values of Boltzmann fits are given in mV.

All values are mean ± S.E.M. (n = 4−8).

Intrinsic inactivation in Shaker channels depends on an N-terminal ball and chain mechanism (N-type) or a K^+^-sensitive conformational change in the pore (C-type) [48], [49], [50]. N-type inactivation has been confirmed in cnidarians in jShak1 [23]. We used N-terminus truncations to examine the mechanism of inactivation in NvShak1,4 and 5 (Figure 2c-e). Truncation removed almost all inactivation from NvShak1 and NvShak5, demonstrating that these channels inactivate primarily by an N-type ball and chain mechanism. Removal of the N-terminus in NvShak4 removed fast inactivation, but unmasked a second slower inactivation process that was K^+^ sensitive. This result suggests that NvShak4 may inactivate by both N-type and C-type mechanisms, similar to the Drosophila Shaker A splice variant [49], and confirms that both mechanisms have been conserved throughout metazoan evolution. Partial inactivation in NvShak6 was not removed by truncation or K^+^ (data not shown).

**Figure 3:**
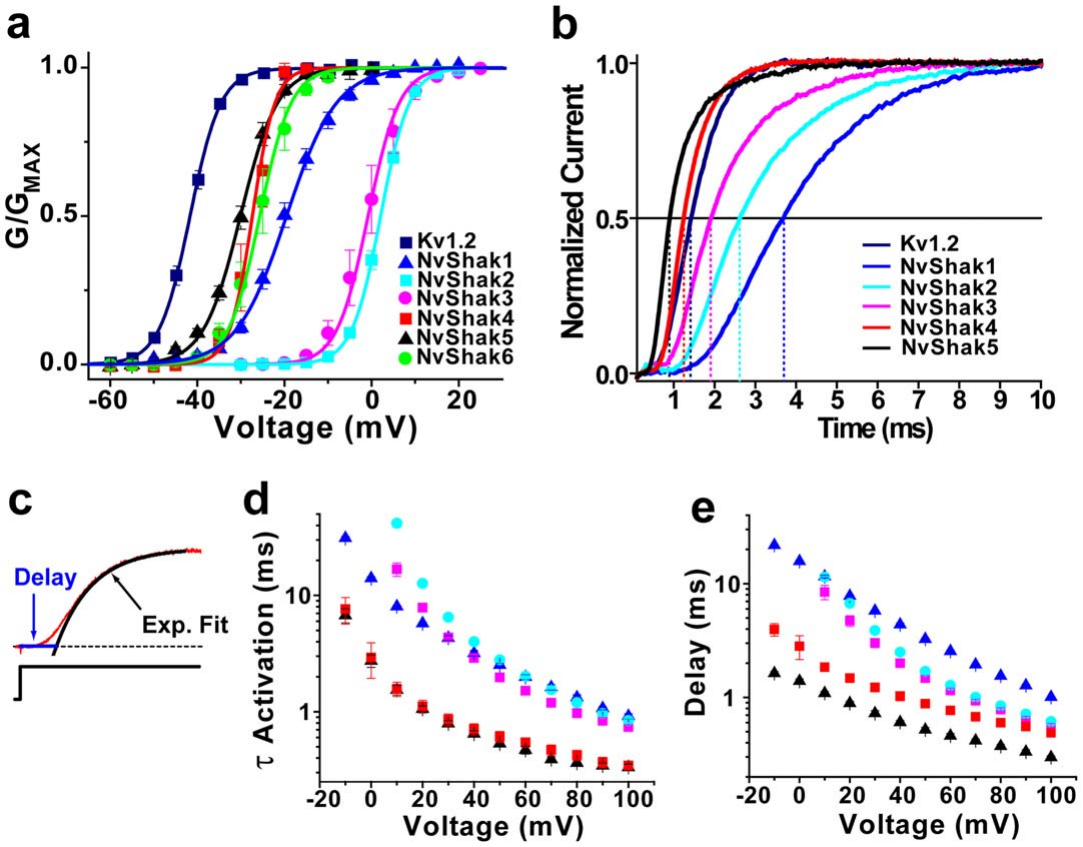
Voltage dependent activation of NvShak currents. *(a)* Voltage activation curves for NvShak1-6 and the mouse Shaker channel Kv1.2. Currents were activated with depolarizing pulses of 100 ms to the indicated voltages from a holding potential of −100 mV, and conductance measurements are taken from isochronal tail currents recorded upon repolarization to −100 mV. Experiments were conducted in symmetrical 140 mM K^+^. Data points are normalized and show mean ± S.E.M. (n = 4−8); curves show single Boltzmann distribution fits; V_50_ and slope are reported in Table 1. ***(b)*** Comparison of current activation time course recorded in response to a voltage step to +60 mV for Kv1.2 and NvShak1-5. Example currents were normalized in amplitude for comparison and truncated versions of NvShak1, NvShak4 and NvShak5 were used to eliminate fast inactivation. The horizontal line marks 0.5 amplitude (half activation), and dotted vertical drop lines are used to mark half-activation times for each current. ***(c)*** Depiction of strategy to quantify activation time course in two parameters: sigmoidal delay and activation time constant. An example current is shown with a red line, current baseline is indicated with a dashed line and the voltage step is marked below. The activation time constant was determined from single exponential fit of the last 50% of the activation time course (black curve) and the delay was defined as the time between the start of the voltage pulse and the point at which the exponential fit intercepted current baseline (blue line). Delay of current occurs because of rate-limiting closed-closed transitions in the activation pathway [52]. Time constants and delay values of activation for steps to the indicated voltages are shown for NvShak1-5 and mouse Kv1.2 in ***d*** and ***e***, respectively. Values show mean ± S.E.M. (n = 4−8); all experiments were carried out on excised patches.

We next examined the voltage dependence of activation gating in NvShak1-6. Voltage-activation curves for NvShak1-6 and mouse Kv1.2 are shown in Figure 3a, and parameters of single Boltzmann fits are given in Table 1. Data were determined from isochronal tail currents recorded following 100 ms depolarizing voltage steps. N-terminal truncated versions of NvShak1, NvShak4 and NvShak5 were used to eliminate N-type inactivation which otherwise obscured activation time course. Currents were recorded in symmetrical 140 mM K^+^ to maximize inward tail currents and to reduce C-type inactivation in NvShak5. We observed two distinct activation phenotypes in the Nematostella channels. One group of channels, consisting of NvShak1 and NvShak4-6 had low activation thresholds and V_50_ values ranging from −20 to −30 mV; this range of voltage activation is similar to (but slightly more depolarized than) that of typical bilaterian Shaker channels such as mouse Kv1.2 (Figure 3a, Table 1). The second group, comprising NvShak2 and NvShak3, activated only at depolarized voltages and had V_50_ values above 0 mV. This high activation threshold is similar to that observed for the cnidarian Shaker channels jShak1 and jShak2 [23]. Thus, Nematostella, and presumably other cnidarians, appear to have two suites of Shaker channels tuned to operate in different voltage ranges. Since Shaker channels are generally believed to serve a direct role in action potential repolarization, we speculate that these two channel types could operate in neurons with distinct spike thresholds. Alternatively, we speculate that they could be used together in single neurons to facilitate distinct high and low threshold spiking behavior, as is observed in giant motor neurons in medusa of Aglantha digitale [51].

The time course of currents recorded in response to +60 mV voltage steps for NvShak1-5 and mouse Kv1.2 is compared in Figure 3b. Half-activation times (dotted lines) ranged from less than 1 ms for NvShak5 to almost 4 ms for NvShak1; mouse Kv1.2 grouped with the fastest Nematostella channels. The activation time course of NvShak6 was not examined in detail because inactivation could not be reduced sufficiently. Shaker channels activate with a rapid but sigmoidal time course; delay in activation is caused by multiple voltage-dependent closed-closed transitions traversed in the activation pathway [52]. We quantified the time course of activation for NvShak1-5 by measuring this delay and fitting a single exponential to the late phase of activation (above half-activation point) (Figure 3c-e). The “fast” channels NvShak4 and NvShak5 had similar time constants for the late phase of activation, but the overall time course of NvShak4 activation was slightly more delayed. The “slow” channels NvShak1-3 also had similar late phase time constants, but the overall time course of NvShak1 was substantially slower at depolarized voltages due to greater delay. These results imply that the diverse activation time courses of NvShak1-5 derive from differences in the rate of both early (delay) and late gating transitions. It should be noted that fast activation is a defining feature of the Shaker channels, and even the activation time course of NvShak1 is rapid when compared to most other voltage-gated K^+^ channels.

**Figure 4:**
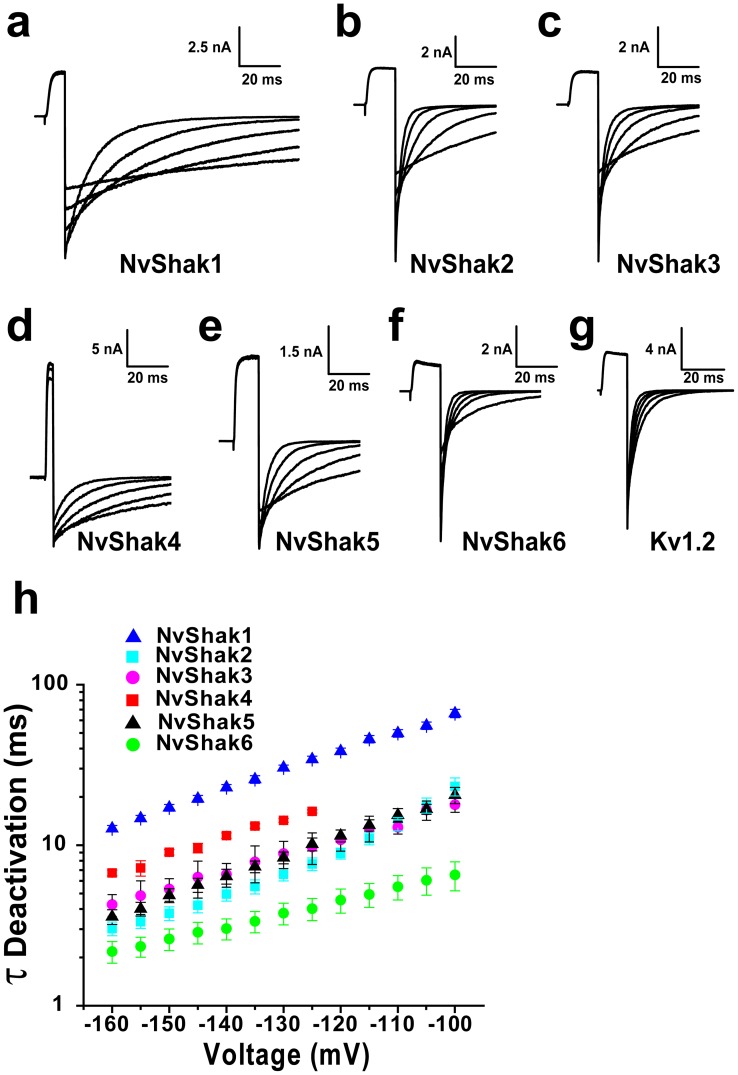
Deactivation of NvShak currents. ***(a–g)*** Tail currents recorded from excised inside-out patches at −160, −140, −120, −100 and −80 mV are shown for NvShak1-6 and mouse Kv1.2. Channels were activated with a test pulse to +50 mV and recordings were made in symmetrical 140 mM K^+^. Scale bars indicate current amplitude and time. ***(h)*** Time constants from single exponential fits of tail current decay are plotted vs. voltage. The slope of the relationship in the semi-logarithmic plot is proportional to voltage-dependence of the closing transition and is qualitatively similar for all channels. Values show mean ± S.E.M. (n = 5−7). NvShak channels displayed ∼10-fold variation in deactivation rate from NvShak6 (fastest) to NvShak1 (Slowest).

We quantified the deactivation rates of NvShak1-6 with single exponential fits of tail current decay. Families of tail currents recorded from patches bathed in symmetrical 140 mM K^+^ are shown for each Nematostella channel and mouse Kv1.2 in Figure 4(a–g). Nematostella Shaker deactivation was typically slower than deactivation of Kv1.2 with the exception of NvShak6. Deactivation time constants for NvShak1-6 are plotted vs. voltage in Figure 4 h. Channels showed a qualitatively similar voltage-dependence for deactivation (slope), but a 10-fold variation in time constant from NvShak6 (fast) to NvShak1 (slow). The rank order of deactivation time constants shown in Figure 4 h differs from the rank order of activation time course (Figure 3c–e); NvShak1-6 thus provide 5 clearly differentiated combinations of kinetics with respect to inactivation, activation and deactivation. Only NvShak2 and NvShak3 are qualitatively similar by all measures.

### Regulatory Subunits

We reasoned that the 12 Nematostella Shaker genes that did not express functional homomultimeric channels *in vitro* might instead encode regulatory subunits that modify the functional properties of NvShak1-6 through co-assembly into heterotetrameric channels. In mammals, the Shab (Kv2) family contains an expansion of 10 genes that do not functionally express as homomultimers [3]. Instead, they co-assemble with Kv2.1 or Kv2.2 subunits (which can assemble as a homomultimers) to produce a palette of delayed rectifier currents with diverse biophysical properties [26], [53], [54], [55], [56], [57]. Similarly the hydrozoan Polyorchis penicillatus has a Shal (Kv4) family regulatory subunit that does not express as a homomultimer but drastically changes inactivation rate and activation threshold in heteromeric Kv4 channels [24]. To test this hypothesis, we co-expressed each of the twelve full length potential regulatory subunits (NvShakRx) with NvShak3 in Xenopus oocytes and looked for novel current properties as an indication for assembly of functional heterotetramers. We chose NvShak3 because it does not have intrinsic inactivation; therefore presence of inactivation in co-expression studies could serve as an indicator for heteromultimer formation.

**Figure 5:**
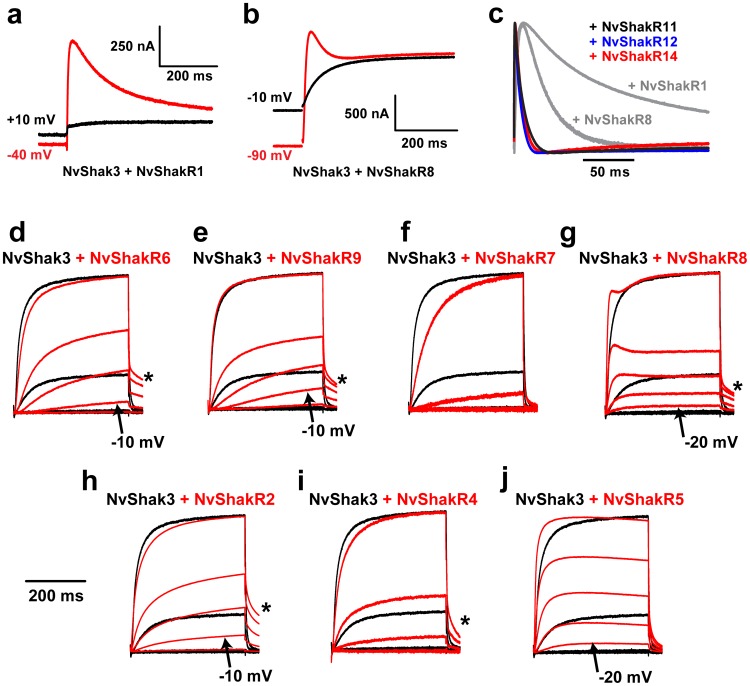
Modification of NvShak3 currents by regulatory NvShakR subunits. Currents elicited by test pulses to +60 mV in whole oocytes co-expressing NvShak3 with NvShakR1 ***(a)*** or NvShakR8 ***(b)*** had inactivating components when preceded by hyperpolarized (red) but not depolarized (black) 5 s pre-pulses. Inactivation during the depolarized pre-pulse removes heteromeric currents and leaves a non-inactivating component that could be contributed by NvShak3 homomers. Scale bars indicate current amplitude and time. ***(c)*** Inactivating components of currents elicited at +60 mV from a −90 mV holding potential from whole oocytes co-expressing NvShak3 with NvShakR11 (black), NvShakR12 (blue) and NvShakR14 (red) are compared. Only the inactivating component (normalized in amplitude) of the currents is shown. Gray lines show the inactivating components from ***a*** (NvShak3/NvShakR1) and ***b*** (NvShak3/NvShakR8) and scale bar indicates time. ***(d–j)*** Co-expression of NvShak3 with seven NvShakR subunits induced changes in activation threshold, activation rate or deactivation rate. Families of currents recorded from whole oocytes under two-electrode voltage clamp in response to 400 ms depolarizations ranging from −20 to +20 mV in 10 mV increments (from a resting potential of −100 mV) are shown for homomeric NvShak3 (black) and NvShak3 co-expressed with the indicated NvShakR subunit (red). Currents were normalized to peak amplitude at +20 mV to facilitate comparison and the time scale bar next to ***h*** is used for ***d–j***. Significant homomeric NvShak3 currents were first seen in the +10 mV step (black control traces); heteromeric currents activating at more hyperpolarized potentials are indicated with arrows and labels for NvShakR2, NvShakR5, NvShakR6, NvShakR7 and NvShakR9. Unusually slow heteromeric tail currents (recorded at −40 mV following depolarization steps) are marked with asterisks for NvShakR2, NvShakR4, NvShakR6, NvShakR8 and NvShakR9. Slow activation induced by co-expression is apparent for NvShakR2, NvShakR6, NvShakR7 and NvShakR9 (***d–f,h***) across at least part of the voltage range.

Five Nematostella Shakers (NvShakR1, NvShakR8, NvShakR11, NvShakR12 and NvShakR14) did indeed introduce an inactivating component when expressed with NvShak3 (Fig. 5a–c). Since we had no means to selectively block the formation or function of homomeric NvShak3 currents, we expected currents resulting from co-expression of NvShak3 with putative regulatory subunits to derive from a mix of homomeric NvShak3 channels and heteromeric NvShak3/NvShakRx channels. We did indeed observe non-inactivating current components in these co-expression experiments that could correspond to homomeric NvShak3 currents. NvShak3/NvShakR1 and NvShak3/NvShakR8 example currents are shown in Figure 5a,b with and without a 5 s depolarizing pre-pulse to induce steady state inactivation, which is not present in homomeric NvShak3 currents. The depolarizing pre-pulses removed transient heteromeric currents, but left a large, non-inactivating current that may in part be contributed by NvShak3 homomers. Figure 5c shows only the inactivating components of the five heteromeric currents normalized to the same amplitude to facilitate comparison of inactivation time course. Heteromers containing NvShakR1 took ∼1 s to inactivate completely at +60 mV (Figure 5a,c), whereas inactivation was complete within 50 ms for heteromers containing NvShakR11, NvShakR12 or NvShakR14 (Figure 5c). Heteromers containing NvShakR8 had an intermediate inactivation time course. Inactivating components were always present upon co-injection of these five regulatory subunits with NvShak3 (n = 4−6). These results demonstrate that combinations of classical NvShak subunits and regulatory NvShakR subunits could provide Nematostella with a palette of inactivating Shaker channels that vary more than 100-fold with respect to inactivation time course (Figure 1, Figure 5a–c, Table 1). We speculate these channels could be used to diversify spike shape and frequency.

Seven of Nematostella Shaker regulatory subunits measurably altered activation and/or deactivation of NvShak3, including NvShakR8. Examples of whole cell currents recorded in response to depolarizing voltage steps (to −20, −10, 0, +10 and +20 mV) are shown for each heteromeric combination compared to homomeric NvShak3 in Fig. 5 d–j. Similar results were always observed during 4–6 independent measurements. NvShak3 currents activated at more depolarized potentials in whole cell two-electrode voltage clamp recordings than in excised patches; the first significant homomeric NvShak3 currents in this protocol appear during the +10 mV step. Five regulatory subunits, NvShakR2, R3, R5, R8 and R9, hyperpolarized the activation range of NvShak3; currents were readily detected at −20 or −10 mV (Fig 5d–g). Currents at these voltages must be purely heteromeric in origin because we never observed current in NvShak3 controls. Currents observed at +10 mV and above probably reflect activity of both heteromeric and homomeric channels. Five regulatory subunits, NvShakR2, R4, R6, R8 and R9 slowed the deactivation rate of currents at −40 mV (asterisks in Figure 5d–j). We also observed unusual slow activation at least at some voltages for NvShakR2, R4, R6, R7 and R9; this was the only affect observed for NvShakR7 (Fig. 5f). A summary of novel heteromeric NvShakRx/NvShak3 current properties is given in Table 2. We were prevented from quantifying the properties of heteromeric currents in detail because of contamination with homomeric NvShak3 currents. Nevertheless, this simple co-expression assay demonstrates that at least 11 Nematostella Shaker subfamily genes encode channels with a regulatory phenotype: they fail to express as homomers but can functionally integrate into heteromeric Shaker channels to regulate biophysical properties.

**Table 2:** Summary of Novel Current Properties in NvShak3 + NvShakRx Heteromers.

	Inactivation	Shift in Threshold	Activation Rate	Deactivation Rate
**NvShakR1**	**Slow**	N.D.	N.D.	N.D.
**NvShakR2**	No	**Medium**	**Slow**	**Slow**
**NvShakR4**	No	**Small**	**Slow**	**Slow**
**NvShakR5**	No	**Large**	**Fast**	No
**NvShakR6**	No	**Medium**	**Slow**	**Slow**
**NvShakR7**	No	No	**Slow**	No
**NvShakR8**	**Fast**	**Large**	N.D.	**Slow**
**NvShakR9**	No	**Medium**	**Slow**	**Slow**
**NvShakR11**	**Very Fast**	N.D.	N.D.	N.D.
**NvShakR12**	**Very Fast**	N.D.	N.D.	N.D.
**NvShakR14**	**Very Fast**	N.D.	N.D.	N.D.

Shift in Threshold is in the hyperpolarizing direction and the magnitude is estimated from the observed voltage at which current first appears (arrows in Figure 5 d–j).

No, the property was not changed in heteromers.

N.D., the property was not determined.

Only one of the putative regulatory subunits, NvShakR10, failed to produce a measurable alteration of NvShak3. Our experiments do not rule out a regulatory functional role for NvShakR10; it may co-assemble with other NvShak subunits or regulate functional properties that we could not easily measure in this biophysical assay. For instance, we would not have detected a shift of activation to more depolarized potentials because of contamination with homomeric NvShak3 currents. NvShakR subunits could alternatively also affect modulation, trafficking or subcellular localization or of Shaker channels. For instance, inclusion of the mammalian Shaker channel subunit Kv1.5 in heteromeric channels confers regulation by Src-family tyrosine kinases [46]. Phosphorylation of neighboring subunits suppresses the heteromeric current. While our current study was limited to detection of biophysical changes, the functional roles of regulatory subunits are potentially far more diverse.

These experiments do not address what heteromeric channel combinations may exist *in vivo*, as these will depend on which subunits are co-expressed and whether NvShakR subunits are able to functionally co-assemble with multiple NvShak1-6 partners. We cannot rule out that co-expression of multiple NvShakR channels together would result in functional channels, but mammalian Shab-related regulatory subunits do not co-assemble into functional channels (data not shown). Nevertheless, combinatorial expression of NvShak1-6 with regulatory subunits could clearly provide a huge diversity of Shaker channel phenotypes in Nematostella.

### Potential Conservation of PDZ-dependent Localization

Most mammalian Shaker genes (Kv1.1-1.7) contain C-terminal PDZ-binding motifs that anchor the channels to synapses and axons through interaction with MAGUK (membrane-associated guanylate kinase) family PDZ proteins such as PSD-95 and PSD-93 [58], [59]. The PDZ-binding motif and PDZ-dependent sub-cellular targeting of Shaker channels in neurons by MAGUK family proteins is conserved in Drosophila and Aplysia [60], [61]. We found archetypal Class I PDZ-binding domain consensus sequences [62] at the C-termini of NvShak2 (-ETLL), NvShak3 (-ETPV) and NvShak4 (-ETGV). Furthermore, the C-termini of NvShak1 (-HTTV), NvShak5 (-QTSV) NvShak6 (-DGFV) and NvShakR10 (-EFTV) differ from this consensus by a single conservative substitution (underlined); each substitution has been observed in a few PDZ-binding motifs [62]. We used BLAST searches to identify a close Nematostella ortholog (JGI protein ID 200894) of the MAGUK family kinases that bind to bilaterian Shaker channels. The predicted protein shares a similar domain structure with PSD-95 and PSD-93 with 3 N-terminal PDZ domains, a central SH3 domain and a C-terminal guanylate kinase domain. Therefore it is possible that at least some Nematostella Shaker channels are clustered at synapses and/or axons in a PDZ-dependent manner, and that this localization mechanism evolved in the first nervous systems.

### Expression Patterns

**Figure 6:**
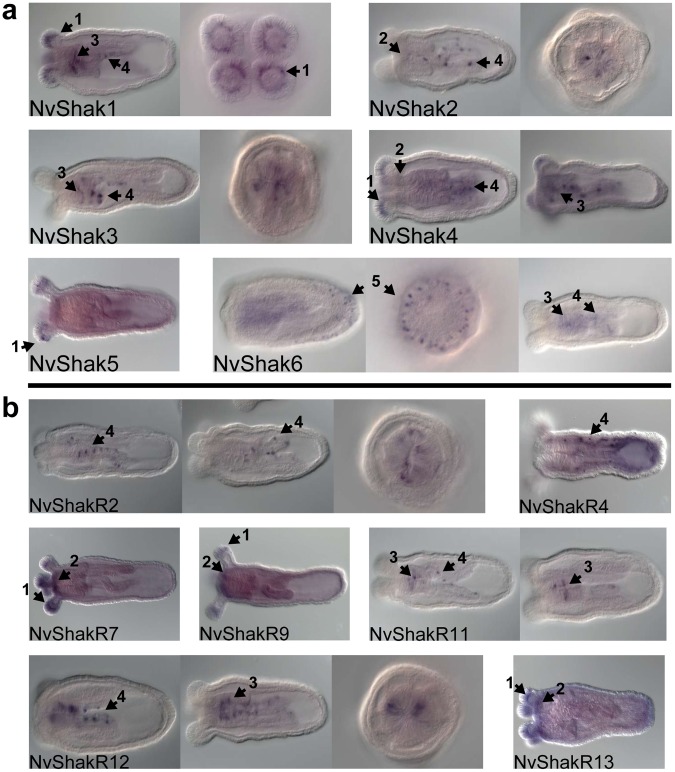
Expression patterns of Nematostella Shaker genes determined by in situ hybridization. Expression in early stage polyps is shown for NvShak1-6 in ***a,*** and 7 NvShakR genes in ***b***. Sagittal views are shown for all probes; coronal views are also shown for NvShak1, NvShak2, NvShak3, NvShak6, NvShakR2 and NvShakR12. Numbered arrows point to select regions of expression. (**1**) Homotetramer-competent inactivating subunits, NvShak1, NvShak4 and NvShak5, are expressed in developing tentacle bulbs, along with regulatory subunits NvShakR7, NvShakR9 and NShakR13. The same three regulatory subunits show light expression along with NvShak2 and NvShak4 in the vicinity of the developing oral nerve ring (**2**). Expression around the pharynx and pharyngeal nerve ring occurs for NvShak1, NvShak3, NvShak4, NvShak6 and the regulatory subunits NvShakR11 and NvShakR12 (**3**). All homotetramer-competent subunits (except NvShak5) and the regulatory subunits NvShakR2, NvShakR4, NvShakR11 and NvShakR12 show expression in subsets of cells in the mesenteries (**4**). NvShak6 expression is concentrated in body cells near the apical pole in early stage polyps (**5**), but transitions to mesenteric and pharyngeal expression as polyp development continues. The specific identity of Shaker-positive cells and cell-level expression overlap of Shaker transcripts remains to be determined.

We examined the expression pattern for 13 Shaker genes in Nematostella polyps using *in situ* hybridization (Figure 6). All were expressed contemporaneously in early stage developing polyps. We observed distinct but potentially overlapping staining patterns that included cells in the tentacles, oral ring, pharynx, mesenteries, and body wall, but we did not establish the functional identity of expression-positive cells. The hypothesis that most Nematostella Shaker genes function as regulatory subunits requires that regulatory subunits (NvShakR1-12) are co-expressed with at least one subunit NvShak1-6 subunit. We did indeed see expression of the NvShak1-6 group in virtually all regions where we also observed expression of an NvShakR subunit (Figure 6). However, we were not able to confirm cell-level co-expression from these *in situ* hybridization results. Therefore, it is not possible for us to determine which homomeric and heteromeric combinations of Shaker channels are used in vivo. Nevertheless, it is clear from preliminary *in situ* analysis that multiple cell populations in Nematostella polyps have the potential to express various combinations of functionally distinct Shaker channel subunits. We suggest that distinct Shaker channel combinations could contribute substantially to diversity of intrinsic electrical properties of neurons and other excitable cells in the Nematostella polyp. Expression patterns are summarized for the 13 Shaker genes in Table S3.

### Phylogenetic Analysis

**Figure 7:**
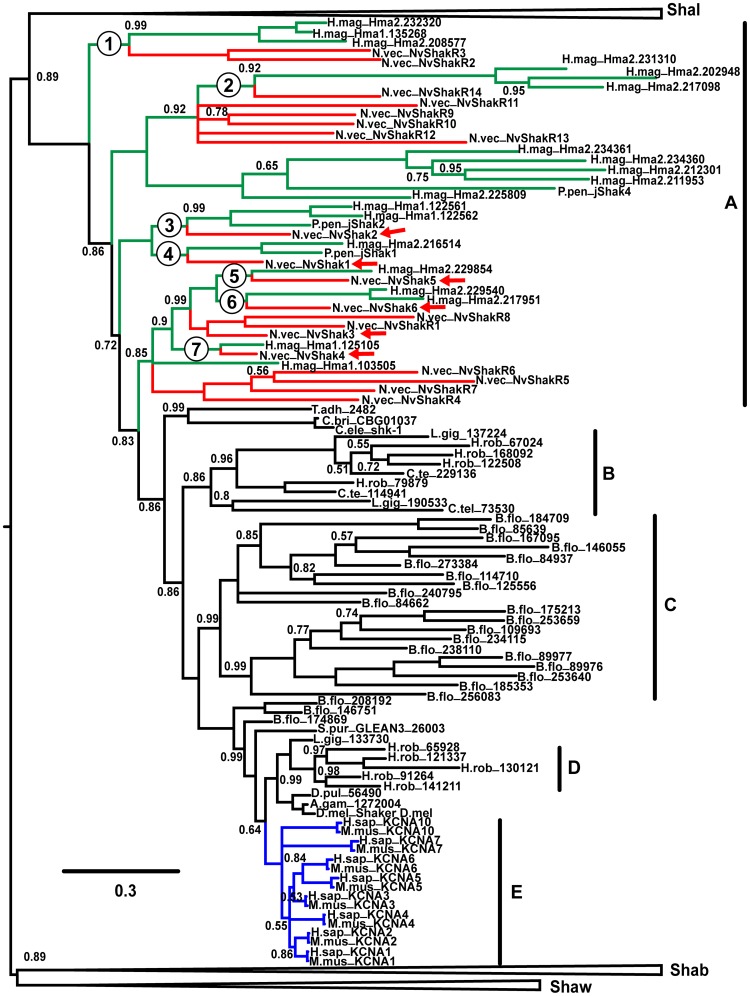
Phylogeny of Metazoan Shaker K^+^ channel subfamily. A Bayesian phylogeny of Metazoan Shaker family is shown with the Shaker subfamily (Kv1) clade expanded; the phylogeny has been midpoint rooted for display purposes. Cnidarian sequences are highlighted in red (Nematostella) or green (Hydra and Polyorchis). Mammalian sequences are highlighted in blue. Circled numbers label seven ancestral cnidarian branches containing both anthozoan and hydrozoan sequences, and red arrows point to NvShak1-6. Bars at the right margin are used to indicate 5 large, species-restricted expansions of Shaker subfamily genes in cnidarians (A), lophotrochozoans (B), amphioxus (C), leech (D) and vertebrates (E). The scale bar indicates number of substitutions/site and posterior probabilities are given at branch points. All unlabeled branches had posterior probabilities equal to 1. Gene names are given at branch ends with a 4-letter prefix to denote species. The prefixes in alphabetical order are A.gam (Anopheles gambiae, mosquito), B.flo (Branchiostoma floridiae, amphioxus), C.bri (Caenorhabditis Briggsae, nematode), C.ele (Caenorhabditis elegans, nematode), C.tel (Capitella teleta, annelid), D.pul (Daphnia pulex, crustacean), D.mel (Drosophila melanogaster, fruit fly), H.rob (Helobdella robusta, leech), H.sap (Homo sapiens, human), H.mag (Hydra magnipapillata, hydra), L.gig (Lottia gigantea, limpet), M.mus (Mus musculus, mouse), N.vec (Nematostella vectensis, sea anemone), P.pen (Polyorchis penicillatus, Hydrozoan jellyfish), S.pur (Strongylocentrotus purpuratus, sea urchin), and T.adh (Trichoplax adhaerens, placozoan). All sequences used in phylogeny construction are listed in Table S1, and expanded views of the Shab (Kv2), Shaw (Kv3) and Shal (Kv4) clades are shown in Figures S1, S2, S3, respectively.

We constructed a Bayesian inference phylogeny of the Shaker K^+^ channel superfamily (including the Shaker (Kv1), Shab (Kv2), Shaw (Kv3) and Shal (Kv4) gene families) using amino acid sequences from mammals, amphioxus, sea urchin, insects, lophotrochozoans, nematodes, trichoplax, and the cnidarians Nematostella, Hydra and Polyorchis. The Shaker subfamily branch of the phylogeny is shown in Figure 7, while the Shab, Shaw and Shal family branches are shown in Figures S1, S2, S3. Sequence of expression-verified clones was used where available. All sequences used in the phylogeny are included in Table S1, and the number of Shaker family genes found in each species is summarized in Table 3. These gene numbers should be used as a lower limit estimate for species with early draft genomes since coverage has gaps; a few additional sequences could conceivably be found in later drafts. We identified 19 Shaker subfamily genes in Hydra (Figure 7), and cnidarian-specific gene expansions were found in the Kv3 and Kv4 families (Figures S2, S3). This indicates that the need for large, diverse sets of Shaker family voltage-gated K^+^ channels is conserved across cnidarian orders. However, we found only a few cases of clear orthology between Nematostella and Hydra channels suggesting that much of the Shaker diversity in these species might therefore derive from separate anthozoan and hydrozoan gene expansions. Only 7 strongly supported branches in the Shaker subfamily tree contain sequences from both species (Figure 7). Interestingly, phylogenetic analysis indicates that 5 of the 6 Nematostella Shakers that express functionally as homomultimers were already present before the anthozoan/hydrozoan split. NvShak4 and NvShak5 have clear 1∶1 orthologs in Hydra. NvShak1, NvShak2 and NvShak6 have clear Hydra co-orthologs, indicating that the genes were present before the anthozoan/hydrozoan split and were subsequently duplicated in the Hydra lineage. Only NvShak3 did not have a clear hydrozoan ortholog. Polyorchis (Hydrozoa) jShak1 and jShak2 are molecular orthologs of NvShak1 and NvShak2, respectively, and show some functional orthology. NvShak1 and jShak1 both exhibit fast N-type inactivation but differ in activation threshold [23]. NvShak2 and jShak2 have similar high activation thresholds, but differ significantly in inactivation rate [23]. The function of these genes has thus diverged modestly in Hydrozoa and Anthozoa since their appearance in ancestral cnidarians.

**Table 3:** Shaker Family Genes in Metazoan Species.

	Shaker (Kv1)	Shab (Kv2)	Shaw (Kv3)	Shal (Kv4)	Total
**Placozoa**					
* Trichoplax Adherens*	1	1	1	0	3
**Cnidaria**					
** Anthozoa**					
* Nematostella Vectensis*	20	1	11	12	44
** Hydrozoa**					
* Hydra magnipapillata*	19	1	13 (9)*	2	35
**Lophotrochozoa**					
** Mollusca**					
* Lottia gigantia*	3	1	13 (10)*	1	18
** Annelida**					
* Helobdella robusta*	13 (9)*	6	7 (3)*	3	29
* Capitella teleta*	3	1	9	1	14
**Nematoda**					
* Caenorhabditis elegans*	1	6	3	1	11
* Caenorhabditis briggsae*	1	6	3	1	11
**Arthropoda**					
** Crustacea**					
* Daphnia pulex*	1	1	3	1	6
** Hexapoda**					
* Drosophila melanogaster*	1	1	2	1	5
* Anopheles gambiae*	1	1	3	1	6
**Echinodermata**					
* Strongylocentrotus purpuratus*	1	1	1	1	4
**Chordata**					
* Branchiostoma floridiae*	23	0	0	19	42
** Vertebrata**					
* Mus musculus*	8	12	4	3	27
* Homo sapiens*	8	12	4	3	27

* Count includes sequences that were not used in the phylogeny because of large gaps. These sequences are included in Table S1 and had reciprocal best BLASTP matches to the subfamily indicated. The number in parentheses indicates the number of sequences included in the phylogeny.

In contrast, it appears that most of the regulatory subunit (NvShakR) diversification occurred after the anthozoan/hydrozoan split. We assumed for this analysis that NvShakR10 has a regulatory phenotype since it does not express as a homomultimer. We also assumed that NvShakR3 and NvShakR13, two incomplete and thus untested clones, also are most likely regulatory subunits because they group closely and exclusively with proven regulatory subunits in the phylogeny. Only two clades exclusively contain Nematostella regulatory subunits and also contain sequences from Hydra. This indicates that the cnidarian-specific regulatory phenotype was present in ancestral cnidarians, but that much of the elaboration of the Nematostella regulatory subunit set occurred after the divergence from Hydrozoa. Late divergence is strongly supported by several instances of separate clustering for Nematostella and Hydra regulatory subunits. However, it is possible that the fast evolutionary divergence observed for regulatory subunits obscures some ancestral relationships between clusters. Cnidarian-specific gene expansions observed in the Shaw and Shal subfamilies also show a high degree of separate duplication in Nematostella and Hydra (Figures S2, S3), and it is therefore tempting to speculate that these expansions may also represent diversification of regulatory subunits. The Polyorchis regulatory subunit jShalγ does indeed fall within a clade that contains a high degree of Nematostella-specific gene duplication. However, confirmation of regulatory phenotype in Nematostella Kv3 and Kv4 subunits awaits functional characterization.

Analysis of the entire Shaker family tree suggests the presence of at least 13 Shaker family genes (7 Shaker, 1 Shab, 3 Shaw and 2 Shal) in ancestral cnidarians (Figure 7, Figures S1, S2, S3). We found 12 supported branches containing both Hydra and Nematostella sequences. A 13^th^ putative ancestral channel in the Shal subfamily is indicated by the close grouping of one Nematostella and one hydrozoan branch with bilaterian sequences separate from other cnidarian sequences. The Polyorchis member of this group is required for functional expression of Polyorchis Shal channels and is functionally orthologous to bilaterian Shal channels [24]. The phylogeny points to a single ancestral gene for each subfamily in the cnidarian/bilaterian ancestor – gene duplication in each phylogenetic group appears to have a separate, more recent history. The phylogeny contains 18 independent expansions of 3 or more genes within a restricted set of species (Figure 7, Figures S1, S2, S3), 5 in Shaker, 3 in Shab, 6 in Shaw and 4 in Shal. For example, cnidarians, amphioxus and mammals all have large expansions in the Shaker subfamily that occurred after their separation from the other species included in the phylogeny. The result of this repeated gene expansion in various species is that almost all the major phylogenetic divisions of extant eumetazoans have highly specialized sets of Shaker family channels.

The presence of Shaker, Shab and Shaw orthologs in the placozoan Trichoplax adhaerens suggests that the Shaker family may predate the origin of true nervous systems. The placozoan genome suggests that these simple animals belong to the eumetaozan clade despite the apparent absence of a nervous system [29]. Genome analysis indicates that many classes of eumetazoan signaling molecules are also present in sponges and may have evolved to enable multicellular communication [63]. We were not, however, able to identify Shaker family genes in the draft genomes of the sponge Amphimedon queenslandica [63] or the choanoflagellate Monosiga brevicollis [64]. Given the phyologenetic diversity of sponges, it remains possible that Shaker channels could be found in other sponge species. At present, however, the Shaker family appears to be uniquely eumetazoan.

### Conclusions

The results presented here show that a high functional diversity of Shaker currents has been preserved throughout eumetazoan evolution. Fast activation, diverse inactivation rates, N-type and C-type inactivation mechanisms and PDZ-dependent clustering may be universal functional properties of the Shaker subfamily within Metazoa. Vertebrates, protostomes and cnidarians all have distinct molecular strategies for producing kinetically diverse Shaker currents. Surprisingly, functional diversity of the Shaker family appears to be highest in cnidarians. Cnidarians have functional orthologs of diverse bilaterian Shaker channels and have so far cnidarian-specific high threshold channels and regulatory subunits. Amphioxus and some lophotrochozoans also have large Shaker gene expansions (Figure 7), but their functional properties have not yet been examined. This unusually high functional diversity in cnidarians may extend to the Shaw (Kv3) and Shal (Kv4) families, which are also highly expanded relative to protostomes and deuterostomes. Phylogenetic analysis shows that gene expansions within the Shaker family have occurred frequently throughout metazoan evolution, including at least three *de novo* emergences of subunits with a regulatory phenotype in the cnidarian Shaker and Shal subfamilies and the vertebrate Shab subfamily. Thus the major metazoan phyla have very unique sets of Shaker family genes.

Our results from studies of the Shaker family indicate that the surprising genomic diversity of voltage-gated ion channels in cnidarians does indeed translate into extensive functional diversity of voltage-gated currents. How this functional diversity contributes to cnidarian neurophysiology is not yet known, but is a topic of considerable interest. Whole genomic analysis suggests the genetic complexity we see in voltage-gated K^+^ channels in cnidarians may be normal rather than an exception [1], [2], [3]. Perhaps extreme functional diversity of neuronal signaling molecules extends the repertoire of behaviors a simple nervous system can generate. Development of efficient genetic tools and neuronal recording techniques for *Nematostella* could enable this question to be answered.

## Supporting Information

Figure S1 **Expanded view of the Shab subfamily tree from the Shaker family phylogeny.** The Shab subfamily clade is shown with Nematostella highlighted in red, Hydra in green and mammals in blue. The circled number labels a single ancestral cnidarian branch and is numbered consecutively relative to the Shaker subfamily clade which contains ancestral branches 1–7 (Figure 7). Bars at the right margin highlight species-restricted expansions of >3 genes and are lettered consecutively with the Shaker subfamily clade (F, leech; G, nematode; H, mammals) which has 5 similar expansions. The scale bar indicates substitutions/site and posterior probabilities are given at branch points only where the value was <1. Gene names are preceded by a species prefix: A.gam (Anopheles gambiae, mosquito), B.flo (Branchiostoma floridiae, amphioxus), C.bri (Caenorhabditis Briggsae, nematode), C.ele (Caenorhabditis elegans, nematode), C.tel (Capitella teleta, annelid), D.pul (Daphnia pulex, crustacean), D.mel (Drosophila melanogaster, fruit fly), H.rob (Helobdella robusta, leech), H.sap (Homo sapiens, human), H.mag (Hydra magnipapillata, hydra), L.gig (Lottia gigantea, limpet), M.mus (Mus musculus, mouse), N.vec (Nematostella vectensis, sea anemone), P.pen (Polyorchis penicillatus, Hydrozoan jellyfish), S.pur (Strongylocentrotus purpuratus, sea urchin), and T.adh (Trichoplax adhaerens, placozoan). Sequences used in phylogenies depicted in the supplemental figures are listed in Table S1.
(PDF)

Figure S2 **Expanded view of the Shaw subfamily tree of the Shaker phylogeny.** Three ancestral cnidarian branches (9–11) are labeled with circled numbers and 6 species-restricted expansions of >3 genes (I, leech; J, arthropods; K, nematodes; L, mammals; M, lophotrochozoans; N, cnidarians) are indicated with bars. Color schemes, branch labels, gene name species codes and scale bar are identical to those used in Figure 7 and Figure S1.
(PDF)

Figure S3 **Full Shal subfamily tree of the Shaker phylogeny.** Two putative ancestral Cnidarian branches (12,13) are labeled with circled numbers and 4 species-restricted expansions of >3 genes(O, mammals; P, leech; Q, amphioxus; R, cnidarians) are indicated with bars. Color schemes, scales, branch labels and gene names follow the conventions of previous phylogeny figures.
(PDF)

Table S1 **Amino acid sequences for metazoan Shaker family channels.**
(XLSX)

Table S2 **DNA sequences for the coding regions of Nematostella Shaker subfamily channels.**
(XLSX)

Table S3 **Summary of Nematostella Shaker expression patterns.**
(PDF)
