# Supplementary figures and images for: Expanded Functional Diversity of Shaker K+ Channels in Cnidarians Is Driven by Gene Expansion
Source: PLoS One. 2012 Dec 10;7(12):e51366. doi: 10.1371/journal.pone.0051366 (PMC3519636; doi:10.1371/journal.pone.0051366)

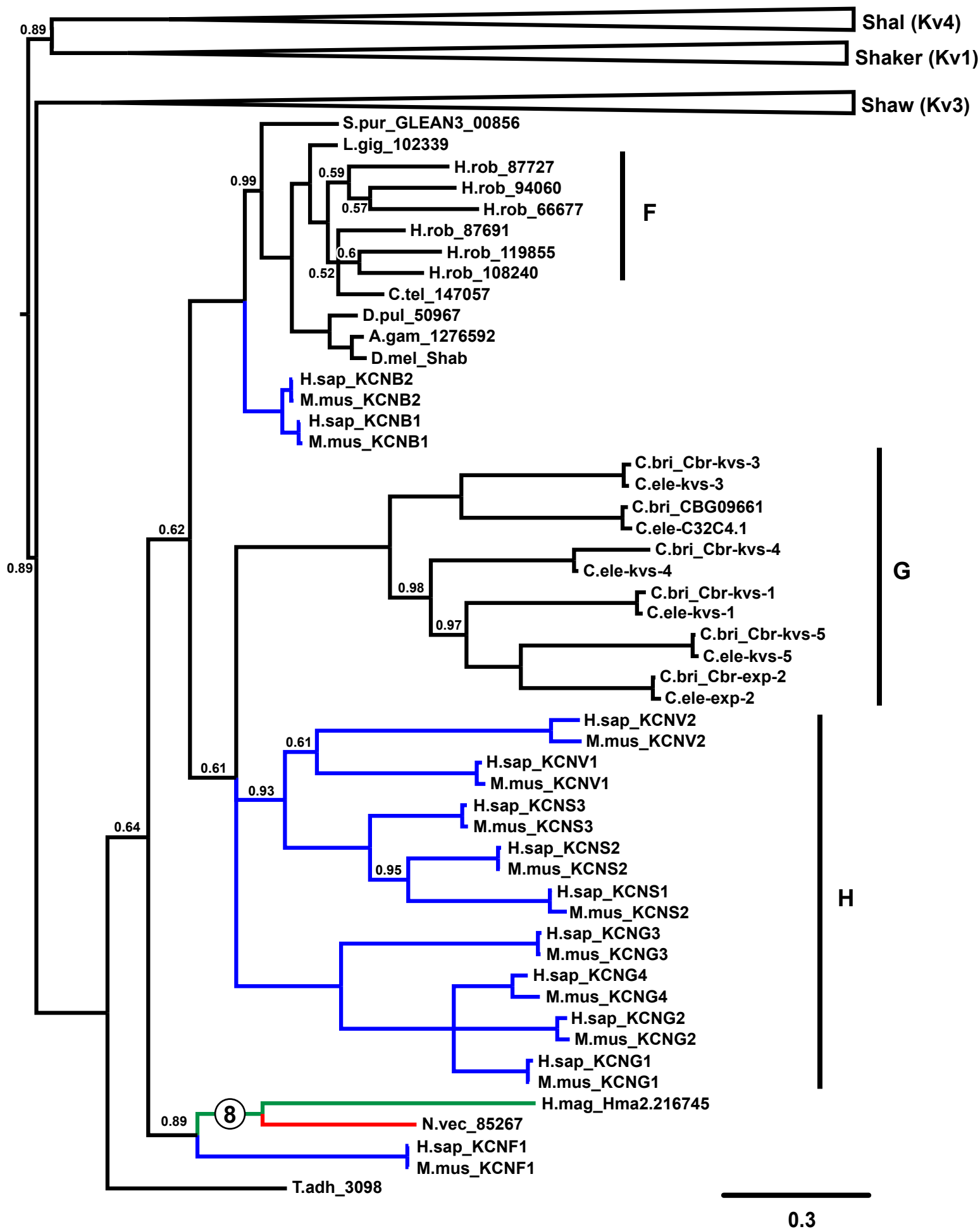

Supplement: Figure S1 — Expanded view of the Shab subfamily tree from the Shaker family phylogeny. The Shab subfamily clade is shown with Nematostella highlighted in red, Hydra in green and mammals in blue. The circled number labels a single ancestral cnidarian branch and is numbered consecutively relative to the Shaker subfamily clade which contains ancestral branches 1–7 (Figure 7). Bars at the right margin highlight species-restricted expansions of >3 genes and are lettered consecutively with the Shaker subfamily clade (F, leech; G, nematode; H, mammals) which has 5 similar expansions. The scale bar indicates substitutions/site and posterior probabilities are given at branch points only where the value was <1. Gene names are preceded by a species prefix: A.gam (Anopheles gambiae, mosquito), B.flo (Branchiostoma floridiae, amphioxus), C.bri (Caenorhabditis Briggsae, nematode), C.ele (Caenorhabditis elegans, nematode), C.tel (Capitella teleta, annelid), D.pul (Daphnia pulex, crustacean), D.mel (Drosophila melanogaster, fruit fly), H.rob (Helobdella robusta, leech), H.sap (Homo sapiens, human), H.mag (Hydra magnipapillata, hydra), L.gig (Lottia gigantea, limpet), M.mus (Mus musculus, mouse), N.vec (Nematostella vectensis, sea anemone), P.pen (Polyorchis penicillatus, Hydrozoan jellyfish), S.pur (Strongylocentrotus purpuratus, sea urchin), and T.adh (Trichoplax adhaerens, placozoan). Sequences used in phylogenies depicted in the supplemental figures are listed in Table S1. (PDF) [file pone.0051366.s001.pdf]

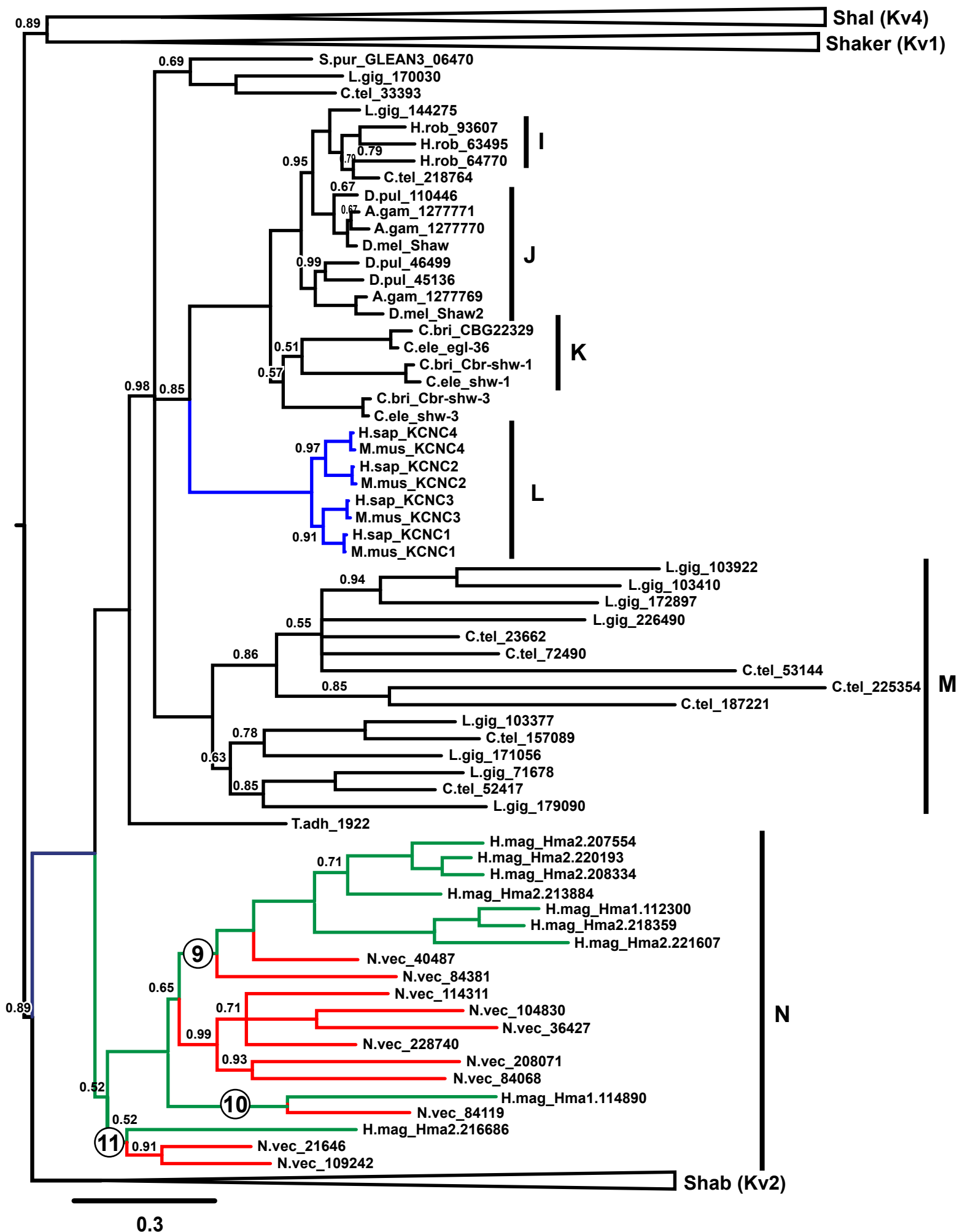

Supplement: Figure S2 — Expanded view of the Shaw subfamily tree of the Shaker phylogeny. Three ancestral cnidarian branches (9–11) are labeled with circled numbers and 6 species-restricted expansions of >3 genes (I, leech; J, arthropods; K, nematodes; L, mammals; M, lophotrochozoans; N, cnidarians) are indicated with bars. Color schemes, branch labels, gene name species codes and scale bar are identical to those used in Figure 7 and Figure S1. (PDF) [file pone.0051366.s002.pdf]

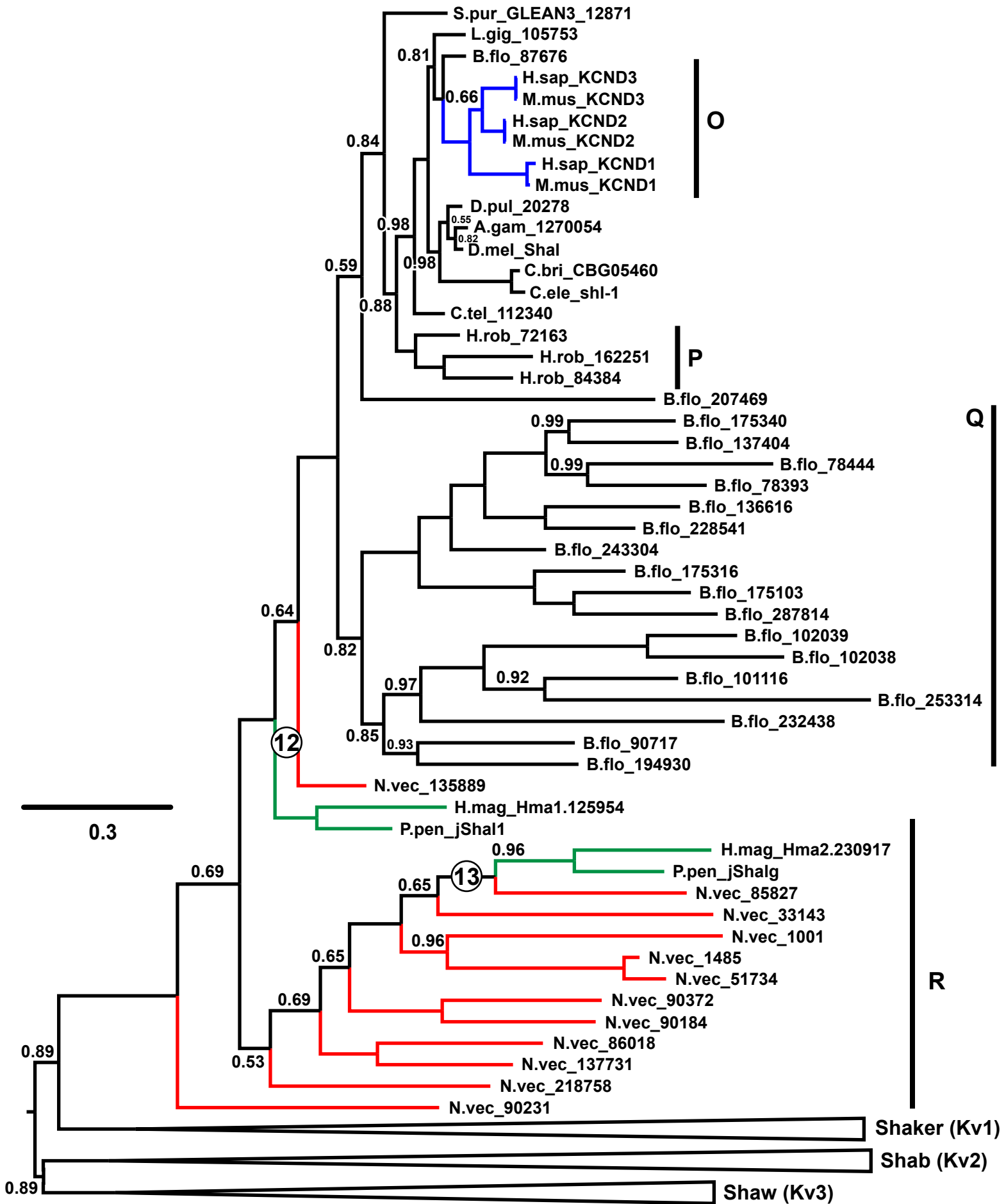

Supplement: Figure S3 — Full Shal subfamily tree of the Shaker phylogeny. Two putative ancestral Cnidarian branches (12,13) are labeled with circled numbers and 4 species-restricted expansions of >3 genes(O, mammals; P, leech; Q, amphioxus; R, cnidarians) are indicated with bars. Color schemes, scales, branch labels and gene names follow the conventions of previous phylogeny figures. (PDF) [file pone.0051366.s003.pdf]
