# Supplementary material for: Expanded Functional Diversity of Shaker K+ Channels in Cnidarians Is Driven by Gene Expansion
Source: PLoS One. 2012 Dec 10;7(12):e51366. doi: 10.1371/journal.pone.0051366 (PMC3519636; doi:10.1371/journal.pone.0051366)
Supplement: Table S3 — Summary of Nematostella Shaker expression patterns. (PDF) [file pone.0051366.s006.pdf]

Table S3. Summary of Nematostella Shaker Expression Patterns

Gene	Tentacle Bulb	Oral Ring	Pharynx Pharyngeal ring	Mesenteries	Apical Wall
NvShak1	X		X	X	
NvShak2		X		X	
NvShak3			X	X	
NvShak4	X	X	X	X	
NvShak5	X				
NvShak6			X	X	X
NvShakR2				X	
NvShakR4				X	
NvShakR7	X	X			
NvShakR9	X	X			
NvShakR11			X	X	
NvShakR12			X	X	
NvShakR13	X	X			

X, expression observed
